# Supplementary material for: Comparative multiomics analysis of cell physiological state after culture in a basket bioreactor
Source: Sci Rep. 2022 Nov 23;12:20161. doi: 10.1038/s41598-022-24687-4 (PMC9686226; doi:10.1038/s41598-022-24687-4)
Supplement: Supplementary file 1 — Supplementary Information 1. [file 41598_2022_24687_MOESM1_ESM.zip › raw data/Proteomic raw data/9.PPI/EG.vs.CG/network_src/network.html]

蛋白质互作分析


:   差异蛋白互作分析


# 差异蛋白互作分析

利用StringDB蛋白质互作数据库(http://string-db.org/)进行鉴定蛋白的互作分析，若在数据库中有相应的物种，则直接提取相应物种的序列，若无，则提取近源物种的序列，然后将差异蛋白的序列与提取出的序列进行blast比对，得出相应的互作信息，构建网络图，网络图的例图见图9。

在结果文件中，提供了蛋白互作数据的文件，可以直接导入 Cytoscape 软件进行可视化编辑，说明文档请**点击**。

排列方式
随机
矩阵
圆形

重置图像
保存图片

图8 蛋白互作网络图

对应的结果目录：蛋白互作结果见：result/9.PPI/\*/\*.Net\_PPI.txt ；
上调蛋白互作结果见：result/9.PPI/\*/\*.Net\_up\_PPI.txt ；
下调蛋白互作结果见：result/9.PPI/\*/\*.Net\_down\_PPI.txt ；
蛋白互作展示图见：result/9.PPI/\*/\*.StringDB\_cut500.{pdf,png}（此图为R包绘制，可用上述Cytoscape的方法制作更美观的网络图）
